# Sleep Quality Among Patients and Healthcare Providers in the Primary Healthcare Setting

**DOI:** 10.3390/jcm14020530

**Published:** 2025-01-15

**Authors:** Rastislava Krasnik, Mirjana Kolundžić, Aleksandra Mikov, Jelena Zvekić-Svorcan, Dragana Vukliš, Milena Kovačević, Andrijana Mikić, Igor Mikov, Dajana Dedić Novaković, Milica Stanić

**Affiliations:** 1 Faculty of Medicine, University of Novi Sad, 21000 Novi Sad, Serbia; rastislava.krasnik@mf.uns.ac.rs (R.K.); aleksandra.mikov@mf.uns.ac.rs (A.M.); jelena.zvekic-svorcan@mf.uns.ac.rs (J.Z.-S.); dragana.vuklis@mf.uns.ac.rs (D.V.); igor.mikov@mf.uns.ac.rs (I.M.); dajana.dedic-novakovic@mf.uns.ac.rs (D.D.N.); milica.stanic@mf.uns.ac.rs (M.S.); 2 Institute of Child and Youth Health Care of Vojvodina, 21000 Novi Sad, Serbia; 3 Health Center “Novi Sad”, 21000 Novi Sad, Serbia; kolundzic.mirjana@dzns.rs; 4 Special Hospital for Rheumatic Diseases, 21000 Novi Sad, Serbia; 5 Faculty of Philosophy, University of Belgrade, 11000 Belgrade, Serbia; andrijana.mikic@ahr.edu.rs; 6 Academy for Human Development, 11000 Belgrade, Serbia; 7 Psychiatry Clinic, University Clinical Center of Vojvodina, 21000 Novi Sad, Serbia; 8 Oncology Institute of Vojvodina, 21204 Sremska Kamenica, Serbia

**Keywords:** Pittsburgh Sleep Quality Index, poor sleep quality, healthcare providers, patients, primary healthcare, sleep

## Abstract

**Background/Objectives:** As adults spend about 30% of each day asleep, having a sleep disorder can negatively affect their functioning. The study objective was to determine the factors that influence sleep quality among patients and healthcare providers in the primary healthcare setting. **Methods:** This study included respondents of both sexes aged 18–90 years, comprising outpatients and the healthcare providers working in the General Medicine Service at the “Liman” Department of the “Novi Sad” Health Center in Novi Sad, Republic of Serbia. Demographic data along with factors related to lifestyle and sleep habits were collected using a demographic questionnaire specifically designed for this research. The standardized Pittsburgh Sleep Quality Questionnaire, Serbian version (PSQI), was used to assess sleep quality. **Results:** The study sample comprised 92 respondents (42 healthcare providers and 50 patients), 73.9% of whom were female, with an average age of 50.0 years. Although 50% of healthcare providers reported sleeping only 3–6 h the previous night, according to the PSQI results, patients had lower overall sleep quality (54.0% vs. 33.3%; *p* = 0.046) and achieved lower scores in the DISTB (sleep disturbances, *p* = 0.001), SLPQUAL (subjective sleep quality, *p* = 0.013) and MEDS (use of sleeping medication, *p* = 0.003) PSQI domains. **Conclusions:** Sleep quality is impaired in more than half of patients and more than a third of healthcare providers. By detecting and acting on the factors in the home and work environment that affect quality of sleep, and by changing lifestyle habits, sleep quality in adults can be improved.

## 1. Introduction

As sleep is an important and necessary part of our lives, quality sleep is crucial for human productivity and health [1,2]. Poor sleep quality can compromise the cognitive processing of information while also increasing the risk of developing depression and anxiety disorders [1,3]. Numerous factors lead to poor sleep quality, especially lifestyle habits, chronic illnesses, and psychological factors. Engaging in regular physical activity can enhance sleep by improving sleep quality, reducing the time it takes to fall asleep, and promoting overall better sleep patterns. Additionally, physical activity has proven beneficial in managing sleep disorders such as insomnia. Moderate-intensity physical activities are the most effective for improving sleep, whereas high-intensity activities, especially in the evening or near bedtime, can disrupt sleep. Other factors that influence the impact of physical activity on sleep quality include gender, age, type of activity, timing, duration, and consistency, all of which also affect overall quality of life, particularly in the adult population [4,5]. However, a growing body of evidence suggests that it can also be caused by socioeconomic factors [6]. Among healthcare providers, the health of their patients also plays a role and thus requires careful examination [1], which can be accomplished with the aid of a variety of scales [7,8,9,10]. Nonetheless, the Pittsburgh Sleep Quality Index (PSQI) [11] is the most widely utilized index in research and practice, as it focuses on different aspects of sleep quality and allows for easier comparison of the results yielded by different studies [12,13,14], whether it is administered independently or in combination with other sleep quality assessment scales [15]. According to our knowledge, no studies have been conducted so far to examine sleep quality among healthcare workers in primary healthcare, nor studies comparing sleep quality between patients and healthcare workers in primary healthcare. Previous studies have focused on quality of sleep among healthcare workers employed in hospital settings [1,2,3], while research conducted on healthcare workers in primary healthcare has primarily focused on issues such as back and neck pain [16,17], rather than on quality of sleep in this population.

The aim of this research was to identify the factors that influence sleep quality in patients and healthcare providers in the primary healthcare setting.

## 2. Materials and Methods

This prospective cross-sectional study was conducted from September 1st to October 1st 2023 and involved adult respondents of both sexes (aged 18 to 90 years). Both outpatients and the healthcare providers working in the General Medicine Service at the “Liman” Department of the “Novi Sad” Health Center in Novi Sad, Republic of Serbia, took part. This study included healthcare professionals (nurses, general practitioners, and specialist physicians), while non-healthcare staff were not included. Considering the study objectives, the sample size calculated with an 80% confidence interval, with a maximum error of 6%, and a critical incidence value of 50%, was 92 respondents.

Participation was voluntary, and all respondents were informed about the objectives and aims of this study. Upon obtaining the approval of the institutional Ethics Committee (Decision number EO: 21/25-1 and 21/23-1) for this investigation, prospective candidates who met the study inclusion criteria signed an informed consent form. They subsequently completed a demographic questionnaire designed specifically for this research, which probed into their age, sex, and educational attainment, as well as factors related to their lifestyle and sleep habits. Their sleep quality was assessed via the standardized Pittsburgh Sleep Quality Questionnaire, Serbian version (PSQI) [18]. Before being included in the study and signing the informed consent form, the examiner asked the participants about the presence or absence of known musculoskeletal disorders. Participants who gave an affirmative answer were not included in the research. Only adults (those aged 18 and above) who provided written informed consent were eligible for participation, while those who reported poor sleep quality due to musculoskeletal system issues and participants with cardiorespiratory, neurological, or psychiatric disorders were excluded, as well as individuals who were unable to complete the questionnaires independently due to cognitive deficits.

When completing the PSQI, the participants were instructed to focus on their sleep quality in the preceding 30 days. To obtain the overall PSQI score, seven components—subjective sleep quality, sleep latency, sleep duration, habitual sleep efficiency, sleep disturbances, use of sleeping medication, and daytime dysfunction—are assessed separately on a 0–3 scale. For each of these components, 0 indicates no difficulty and 3 denotes severe difficulty. Accordingly, the total PSQI score can range from 0 to 21, whereby a higher score is indicative of poorer sleep quality (PSQI ≥ 5 is associated with impaired sleep) [11]. PSQI is a reliable and valid instrument that has been extensively used in academic research and clinical practice. It has been translated into several languages, and the Serbian version has a sensitivity of 70.5% and a specificity of 71.9% [18,19].

### Statistical Analysis

Due to the nature of the demographic questionnaire and the PSQI instrument, the obtained data were subjected to descriptive analyses. The results pertaining to categorical variables were presented as frequencies and percentages, while median and interquartile range or mean and standard deviation were reported for continuous variables. The Mann–Whitney U test was performed to assess differences between continuous variables, while the Likelihood Ratio test was used for categorical variables. To examine the impact of group affiliation, sociodemographic characteristics (as the independent variables), and sleeping habits on the PSQI Total Score (as the dependent variable), we performed a series of univariate linear regression models. The effect estimates were presented as Beta (β) coefficients, with a corresponding 95% confidence interval (95% CI). The probability level of *p* ≤ 0.05 was considered statistically significant. All statistical analyses were carried out using IBM SPSS Statistics for Windows, ver. 24.0 (IBM Corp., Armonk, NY, USA).

## 3. Results

**Figure 1:**
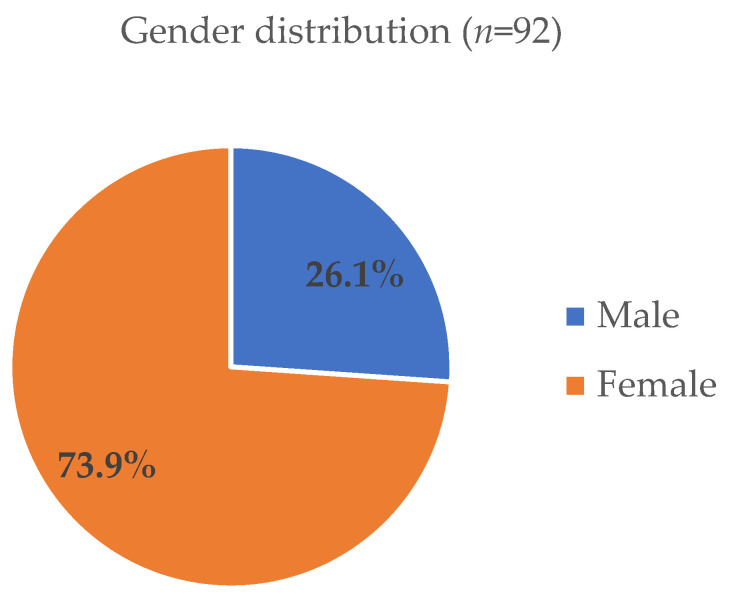
Gender distribution of participants.

**Figure 2:**
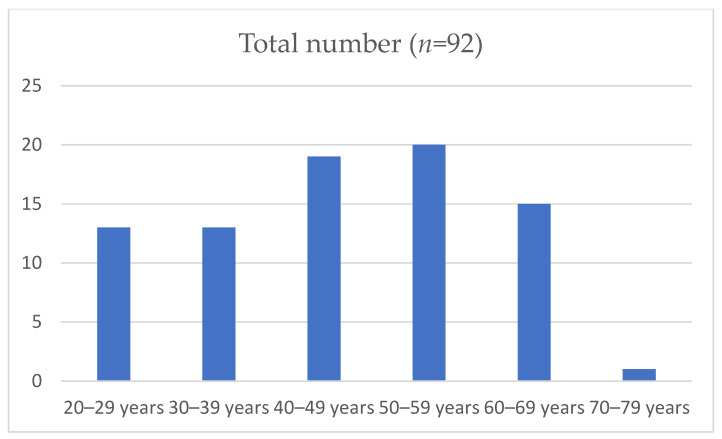
Distribution of participants by age decades.

The study sample included 92 respondents (42 of whom were healthcare providers and 50 were patients) with an average age of 50.0 years (Mdn = 50.0, IQR = 21.0). Moreover, 73.9% of the participants were female, 88.0% lived in the city, and 50% were educated at the secondary school level (Figure 1 and Figure 2). The household size (i.e., the number of family members with whom respondents lived) ranged from 1 to 6 (Mdn = 3.0, IQR = 2.0).

The participants had an average length of employment of 21 years (Mdn = 21.0, IQR = 24.0). Half of the healthcare providers slept 3–6 h the previous night, while the other half slept more than 6 h. When responding to this same question, 14.0% of patients chose <3 h, 42.0% selected 3–6 h, and 44.0% reported >6 h, indicating overall shorter sleep duration compared to the healthcare providers (*p* = 0.011).

Energy drink consumption was reported by 7.1% of healthcare providers and 4.0% of patients (*p* = 0.508), while over 85% of both groups consumed coffee (*p* = 0.746). Although a slightly higher percentage of patients (26.0%) compared to healthcare providers (16.7%) reported regularly taking part in recreational physical activities, the difference was not statistically significant (*p* = 0.276). On the other hand, sleeping with the TV on was more prevalent among patients (18.0%) than healthcare providers (2.4%) and this difference was statistically significant (*p* = 0.010). A comparable percentage of healthcare providers (4.8%) and patients (4.0%) sleep with the light on (*p* = 0.859). Although a higher percentage of patients get up at night (46%) compared to healthcare providers (31%), this difference is not statistically significant either (*p* = 0.139).

Need to use the toilet (77.8%) was the most common reason for getting up during the night, followed by taking care of a small child (11.1%), insomnia (8.3%), and leg cramps (2.8%). Although a smaller percentage of healthcare providers (2.4%) relative to patients (12%) consumed herbal sleep remedies, the difference was not statistically significant (*p* = 0.066). On the other hand, a significantly higher percentage of patients (26%) compared to healthcare providers (2.4%) took sleeping medications (*p* = 0.001), as shown in Table 1.

The differences between healthcare providers and patients regarding various aspects of sleep and overall sleep quality, as measured by the PSQI scale, are shown in Table 2. As previously noted, a total score ≥ 5 on the PSQI scale indicates impaired sleep quality, and this value was recorded in a statistically significantly greater percentage of patients (54.0%) compared to healthcare providers (33.3%), *p* = 0.046.

Based on their scores related to the different PSQI components, patients have worse sleep quality compared to healthcare providers in the following domains: DISTB (sleep disturbances), M = 1.15 (SD = 0.59) vs. M = 0.93 (SD = 0.41), *p* = 0.001; SLPQUAL (subjective sleep quality), M = 0.93 (SD = 0.68) vs. M = 0.74 (SD = 0.59), *p* = 0.013; and MEDS (use of sleeping medication), M = 0.50 (SD = 1.00) vs. M = 0.17 (SD = 0.58), *p* = 0.003.

Table 3 shows the results yielded by the univariate linear regression models with PSQI Total Score as the dependent variable. It is evident that patients have poorer sleep quality (β = −0.28; 95% CI [−3.14, −0.55]; *p* < 0.01), whereby group affiliation explains 7% of the variance in the dependent variable. The following independent variables also emerged as the predictors of sleep quality: getting up at night (β = 0.43; 95% CI [1.57, 4.07]; *p* < 0.01) and the use of sleeping medication (β = 0.65; 95% CI [4.40, 7.26]; *p* < 0.01).

## 4. Discussion

Sleep is regarded as a period of rest for both the body and mind, during which willpower and consciousness are partially or entirely inactive, and bodily functions are partially reduced [20,21,22,23]. Sleep is one of the basic human physiological needs that needs to be met daily for optimal functioning [24]. Recent research provides new insights into the regions of the central nervous system, their interactions, and functioning during all phases of sleep, as well as the external factors that influence sleep, in light of current theories [25]. While most adults require about eight hours of sleep per night, lifestyle differences and other factors, especially psychosocial stressors, can affect sleep patterns and compromise sleep quality [26,27,28]. In adults, sociodemographic factors often also play a role, leading to poor sleep quality (PSQI ≥ 5) and shorter sleep duration (<7 h/night). Based on the survey of 464 working adults (79.5% of whom were men, with an average age of 39 years) conducted in Singapore, 66.2% of respondents slept less than 7 h/night and 42.5% reported having poor sleep quality, which was attributed to age, chronic diseases and conditions, mental health, stress at home or at work, and shiftwork [29]. Similar findings were obtained in a study involving 165,193 members of the general population, as poor sleep quality was linked to poor sociodemographic status (illiteracy, low income, and unemployment), harmful habits and resulting health outcomes (smoking, high-risk drinking, sedentary lifestyle, obesity, diabetes, and hypertension), and poor mental health (perceived poor health status, stress, depressive symptoms, and subjective cognitive decline) [30].

In our research, half of the healthcare providers did not sleep enough the night before (3–6 h), but the patients reported a statistically significantly shorter sleep duration (*p* = 0.011). This finding counters previous reports indicating that sleep problems are four times more common among medical personnel than in the general population [31,32]. While this pattern has been recently ascribed to the COVID-19 pandemic as the need for shiftwork and consecutive shifts increased dramatically, leading to greater burnout [33,34,35,36], this problem was present among healthcare providers prior to the pandemic and is still common even though healthcare systems have returned to their normal mode of operation [37,38].

Sleep difficulties are also related to the level of healthcare that health professionals provide to patients. For example, according to a study conducted among healthcare workers during the COVID-19 pandemic in China, nurses had significantly worse sleep quality and insomnia compared to doctors [8]. In our research, we analyzed quality of sleep among healthcare providers in the primary care setting, who work in shifts, but never during the night. Nonetheless, the participants still reported impaired sleep quality, which can be attributed to their very high workload, given that a large number of patients are treated on an outpatient basis and require a wide range of health services. Available evidence indicates that healthcare providers employed at the secondary and tertiary levels (i.e., medical personnel working in hospitals) also have difficulty sleeping [39]. In a study conducted in 2023 in Sweden by Güngördü and colleagues, according to the PSQI and the Swedish Workload–Control–Support Scale scores, 27.2% of hospital administrative staff had poor sleep quality. Shift workers were found to be 1.73 times more likely to have poor sleep quality, while a one-unit increase in job stress increased the risk of poor sleep quality by 2.59-fold [40].

Based on the results reported by Tian et al. in 2022, who analyzed a wide range of sociodemographic, professional, and personal factors related to sleep quality among medical staff in China, based on the survey responses provided by 3684 participants (84.9% of whom were female), 57.9% of the sample was affected by poor sleep quality. Further analyses revealed that poor sleep quality was associated with lower educational attainment, greater need for hospital care, greater number of work hours per week, mobile phone use for more than 30 min before bedtime, shiftwork, and lack of regular exercise [1]. More recently, Hui-Ren et al. analyzed quality of sleep among nurses and determined that the number of night shifts, lack of family support, and compromised health were the main contributors to poor sleep quality [38].

Sleep quality can be impaired by a large number of factors, as shown by Zhang and colleagues whose 2017 study involved 1563 respondents aged 45+ years. While the overall prevalence of poor sleep quality was 20.67%, the percentage was greater among those with a lower level of education, unmarried, and physically inactive, as well as among participants who suffered from both current and chronic diseases. However, physical health emerged as the main determinant of sleep quality in this cohort [41].

In our research, a slightly higher percentage of patients engaged in recreational physical activities compared to healthcare providers (26.0% vs. 16.7%). In 2022, Tian et al. conducted a survey among healthcare providers and similarly found an insufficient level of regular physical activity among the study participants. The authors thus recommended the use of regular work breaks and better overtime scheduling, along with the adoption of exercise programs, relaxation training, and stress management initiatives as a means of improving the sleep quality of medical personnel [1]. In a survey conducted in Greece, in which 204 doctors and nurses (71.3% of whom were women) took part, a correlation was established between the level of physical activity at work and the amount of free time with the parameters related to sleep disorders [42].

Our investigations revealed that over 85% of both healthcare providers and patients consumed coffee, which could have contributed to lower sleep quality [43], while the consumption of energy drinks was less common. Sleep quality can also be compromised by sleeping with the TV or light on, but these poor habits were not highly prevalent in our cohort (TV: 18% vs. 2.4%, *p* = 0.010; light: 4.8% vs. 4.0%, *p* = 0.859). However, a large percentage of healthcare providers (46%) as well as patients (31%) regularly got up during the night (*p* = 0.139). As the patients were on average older than the healthcare providers, this finding is expected, as older individuals tend to have fewer hours of continuous quality sleep and typically need to use the toilet during the night, especially in the early morning hours.

The use of television, mobile phones, and other distractions can significantly compromise a person’s capacity to fall asleep and attain uninterrupted quality sleep. A growing body of evidence indicates that excessive use of a mobile phone before going to bed can adversely affect sleep quality, thus increasing the risk of difficulties in cognitive functioning during the day [44]. Similar findings relate to watching TV close to bedtime. For example, based on their study conducted in 2016—in which 1500 members of the general population aged 15–90 years took part—Exelmans and colleagues concluded that women had worse sleep quality compared to men. In addition, watching TV for more than three hours and using a computer or mobile phone for more than four hours increased the likelihood of poor sleep quality, as determined by the PSQI scores [7]. Research also shows that prolonged mobile phone use before bedtime is associated with earlier rising and shorter sleep duration among older individuals [9].

Although our analyses revealed different factors that contribute to sleep disturbances, getting up during the night to use the toilet (77.8%) was most common, followed by waking up to take care of a small child (11.1%), insomnia (8.3%), and leg cramps (2.8%). Patients needed to use the toilet at night more often than healthcare providers, which can be attributed to their older age, as noted earlier. However, even younger individuals can suffer from sleep disturbances due to their work and family commitments. In particular, the quality of parental sleep is related to their children’s sleep patterns, as was shown by Lee et al. who found that mothers of children with impaired neuromotor development had short sleep duration (nearly 40% slept < 7 h per night), woke up on average 2.2 times per night, and had poor sleep quality (based on an average total PSQI score of 7.9) [45].

In our research, a PSQI score ≥ 5, which indicates impaired sleep quality, was recorded in a statistically significantly higher number of patients compared to healthcare providers, even though a greater percentage of patients took sleeping medications. Similar findings have been obtained in other studies as a part of which the sleep quality of various professionals was assessed, confirming that compromised sleep and reliance on medications can be a significant diagnostic and therapeutic challenge for healthcare professionals, especially those working in primary care settings [1,29,46]. For example, in 2020, Deng and colleagues found that impaired sleep quality among healthcare providers is often associated with workplace stress levels [47]. Changes in social circumstances or daily routine, as well as illness, fear, and anxiety, can have a negative effect on the quality of sleep in the general population as well, as confirmed by a study conducted in Bosnia and Herzegovina, Croatia and Serbia among young adults during the third wave of the pandemic. According to the responses provided by 1058 individuals (81.4% of whom were women), half of the sample reported changes in sleep patterns, while 47.3% slept less than usual, and 8.6% used sleeping pills to ensure sufficient sleep duration [10]. We must not overlook the period of the COVID-19 pandemic, which also led to various sleep disorders [48]. As quality of sleep in adults is typically affected by a complex combination of multiple factors, these need to be identified and mitigated to prevent long-term impairments in quality of life.

Primary healthcare in our country’s healthcare system serves as the first point of contact for patients and bears a significant burden in terms of the number of patients and services compared to the number of practitioners. Healthcare workers in this setting operate in two shifts and on weekends but without night shifts. The demanding workload, combined with a large number of patients, can be an additional source of stress for healthcare workers, potentially affecting their leisure time and disrupting their normal work–rest rhythm, which may ultimately negatively impact their quality of sleep. This study was conducted among service users and healthcare workers in the largest primary healthcare center in our country and the fourth largest in Southeast Europe. According to data from 2016, a total of 963,208 curative and preventive examinations were provided by the General Medicine Service at the “Novi Sad” Health Center, Serbia. A total of 289,676 adult residents relied on primary healthcare services provided by our departments, highlighting the significant workload faced by healthcare workers in primary healthcare [49]. To the best of our knowledge, there is a lack of research on quality of sleep among healthcare workers in primary healthcare within the context of our state healthcare system, which differs from other healthcare systems in Europe.

When interpreting the findings reported herein, it is important to note several limitations of our study, one of which is its short duration. In addition, we did not analyze the level of stress and depression among healthcare providers and patients and did not account for the comorbidities that can affect quality of sleep. Likewise, we did not probe into mobile phone and computer use before going to sleep in our questionnaire, even though these habits are known to affect sleep quality. We also did not analyze the influence of nutrition, despite studies suggesting that certain dietary patterns and specific foods may play a role in promoting high-quality sleep [50].

Nonetheless, the obtained results clearly indicate the need to develop and implement programs aimed at healthcare providers and patients that would promote healthy lifestyles, as well as greater participation in individual and/or group physical activities.

## 5. Conclusions

Sleep quality is impaired in more than half of patients and more than a third of healthcare providers in the primary healthcare setting. By detecting and acting on the factors that affect quality of sleep in the home and work environment, along with making appropriate changes in lifestyle habits, sleep quality among adults can be improved.

## Figures and Tables

**Table 1:** Demographic and sleep-related characteristics of the study population according to group affiliation.

Respondent Characteristics	ALL (*n* = 92)	Healthcare Providers (*n* = 42)	Patients (*n* = 50)	*p*-Value
Sex:				<0.001 ^a^
Male	24 (26.1%)	2 (4.8%)	22 (44.0%)	
Female	68 (73.9%)	40 (95.2%)	28 (56.0%)	
Age (years), Mdn (IQR), Range	50.0 (21.0), 20–87	43.0 (19.0), 20–65	53.0 (25.7), 21–87	0.001 ^b^
Living environment:				0.053 ^a^
Urban	81 (88.0%)	34 (81.0%)	47 (94.0%)	
Rural	11 (1.02%)	8 (19.0%)	3 (6.0%)	
Highest level of formal education:				0.208 ^a^
Secondary school	46 (50.0%)	18 (42.9%)	28 (56.0%)	
Post-secondary school	21 (22.8%)	12 (28.6%)	9 (18.0%)	
University	17 (18.5%)	10 (23.8%)	7 (14.0%)	
Master’s	8 (8.7%)	2 (4.8%)	6 (12.0%)	
Length of employment (years), Mdn (IQR), Range	21.0 (24.0), 0–40	17.5 (26.2), 1–38	26.0 (20.0), 0–40	0.022 ^b^
Household size, Mdn (IQR), Range	3.0 (2.0), 1–6	3.0 (2.0), 1–5	2.0 (1.7), 1–6	0.199 ^b^
Sleep duration the previous night (hours):				0.011 ^a^
<3	7 (7.6%)	0 (0.0%)	7 (14.0%)	
3–6	42 (45.7%)	21 (50.0%)	21 (42.0%)	
>6	43 (46.7%)	21 (50.0%)	22 (44.0%)	
Energy drink consumption:				0.508 ^a^
Yes	5 (5.4%)	3 (7.1%)	2 (4.0%)	
No	87 (94.6%)	39 (92.9%)	48 (96.0%)	
Participation in recreational physical activities:				0.276 ^a^
Yes	20 (21.7%)	7 (16.7%)	13 (26.0%)	
No	72 (78.3%)	35 (83.3%)	37 (74.0%)	
Coffee consumption:				0.746 ^a^
Yes	80 (87.0%)	36 (85.7%)	44 (88.0%)	
No	12 (13.0%)	6 (14.3%)	6 (12.0%)	
Daily coffee consumption (no. of cups), Mdn (IQR), Range	2.0 (2.0), 1–5	2.0 (1.0), 1–3	2.0 (2.0), 1–5	0.375 ^b^
Sleeping with the TV on:				0.010 ^a^
Yes	10 (10.9%)	1 (2.4%)	9 (18.0%)	
No	82 (89.1%)	41 (97.6%)	41 (82.0%)	
Sleeping with a light on:				0.859 ^a^
Yes	4 (4.3%)	2 (4.8%)	2 (4.0%)	
No	88 (95.7%)	40 (95.2%)	48 (96.0%)	
Getting up during the night:				0.139 ^a^
Yes	36 (39.1%)	13 (31.0%)	23 (46.0%)	
No	56 (60.9%)	29 (69.0%)	27 (54.0%)	
Reason for getting up:				0.008 ^a^
Toilet use	28 (77.8%)	9 (69.2%)	19 (82.6%)	
Caring for a small child	4 (11.1%)	4 (30.8%)	0 (0.0%)	
Leg cramps	1 (2.8%)	0 (0.0%)	1 (4.3%)	
Insomnia	3 (8.3%)	0 (0.0%)	3 (13.0%)	
Use of herbal sleep remedies:				0.066 ^a^
Yes	7 (7.6%)	1 (2.4%)	6 (12.0%)	
No	85 (92.4%)	41 (97.6%)	44 (88.0%)	
Use of sleeping medications:				0.001 ^a^
Yes	14 (15.2%)	1 (2.4%)	13 (26.0%)	
No	78 (84.8%)	41 (97.6%)	37 (74.0%)	

Note: ^a^ Likelihood Ratio test; ^b^ Mann–Whitney U test.

**Table 2:** Sleep quality in the patient and healthcare provider groups.

PSQI Subscales and Total Score	Full Sample		Healthcare Providers		Patients		*p*-Value
	Mdn (IQR)	M (SD)	Mdn (IQR)	M (SD)	Mdn (IQR)	M (SD)	
HSE	0.00 (0.00)	0.02 (0.14)	0.00 (0.00)	0.00 (0.00)	0.00 (0.00)	0.01 (0.10)	0.359 ^a^
DURAT	1.00 (1.00)	0.88 (0.90)	1.00 (1.25)	0.86 (1.00)	1.00 (1.00)	0.87 (0.94)	0.693 ^a^
LATEN	1.00 (1.00)	0.94 (0.87)	1.00 (1.00)	0.76 (0.85)	1.00 (2.00)	0.86 (0.86)	0.283 ^a^
DISTB	1.00 (0.00)	1.34 (0.66)	1.00 (0.00)	0.93 (0.41)	1.00 (1.00)	1.15 (0.59)	0.001 ^a^
DAYDYS	0.00 (1.00)	0.62 (0.88)	0.00 (1.00)	0.38 (0.66)	0.00 (1.00)	0.51 (0.79)	0.227 ^a^
SLPQUAL	1.00 (0.00)	1.10 (0.71)	1.00 (1.00)	0.74 (0.59)	1.00 (0.00)	0.93 (0.68)	0.013 ^a^
MEDS	0.00 (0.00)	0.78 (1.18)	0.00 (0.00)	0.17 (0.58)	0.00 (1.25)	0.50 (1.00)	0.003 ^a^
PSQITOT	4.00 (4.00)	5.68 (3.63)	5.00 (3.25)	3.83 (2.39)	3.00 (5.00)	4.84 (3.24)	0.013 ^a^
PSQI ≥ 5, *n* (%)	41 (44.6%)		14 (33.3%)		27 (54.0%)		0.046 ^b^

Note: ^a^ Likelihood Ratio test; ^b^ Mann–Whitney U test; M = mean, SD = standard deviation; Mdn (IQR) = median (interquartile range). Abbreviations: HSE = habitual sleep efficiency, DURAT = sleep duration, LATEN = sleep latency, DISTB = sleep disturbances, DAYDYS = daytime dysfunction, SLPQUAL = subjective sleep quality, MEDS = use of sleeping medication, and PSQITOT = Pittsburgh Sleep Quality Index Total.

**Table 3:** Univariate linear regression model of the associations that group status, sociodemographic variables, and sleeping habits have with sleep quality.

	Beta [95% CI]	Adj. R^2^
Group (ref.: Patents)		0.07
Healthcare providers	−0.28 [−3.14, −0.55] **	
Sex (ref.: Female)		0.01
Male	0.12 [−0.63, 2.42]	
Age (cont.)	0.17 [−0.01, 0.08]	0.02
Living environment (ref.: Rural)		
Urban	0.01 [−2.06, 2.10]	
Educational attainment (ref.: Secondary school)		0.02
Post-secondary	−0.028 [−1.93, 1.50]	
University	0.09 [−1.11, 2.58]	
Masters	0.01 [−2.36, 2.63]	
Length of employment (cont.)	−0.04 [−0.08, 0.07]	0.01
Household size (cont.)	−0.08 [−0.82, 0.35]	0.04
Energy drink consumption (ref.: No)		0.02
Yes	0.10 [−1.52, 4.40]	
Participation in recreational physical activities (ref.: No)		0.02
Yes	0.16 [−0.38, 2.84]	
Coffee consumption (ref.: No)		0.01
Yes	0.02 [−1.80, 2.20]	
Sleeping with the TV on (ref.: No)		0.02
Yes	0.13 [−0.85, 3.46]	
Sleeping with a light on (ref.: No)		0.01
Yes	0.09 [−1.81, 4.77]	
Getting up during the night (ref.: No)		0.174
Yes	0.43 [1.57, 4.07] **	
Use of herbal sleep remedies (ref.: No)		0.01
Yes	0.08 [−1.58, 3.48]	
Use of sleeping medications (ref.: No)		0.42
Yes	0.65 [4.40,7.26] **	

Note. Values represent Beta coefficients with corresponding 95% confidence intervals (CIs); Adj. R^2^ = adjusted coefficient of determination; ** *p* < 0.01.

## Data Availability

The data presented in this study are available on request from the corresponding author.
